# Fetal Head Position during the First Stage of Labor: Comparison between Vaginal Examination and Transabdominal Ultrasound

**DOI:** 10.1155/2014/314617

**Published:** 2014-03-27

**Authors:** Jyothi Shetty, Vinod Aahir, Deeksha Pandey, Prashanth Adiga, Asha Kamath

**Affiliations:** ^1^Department of OBG, KMC, Manipal University, Manipal 576104, India; ^2^Department of OBG, Women and Child Block (WCB), Manipal, Karnataka 576104, India; ^3^Department of Community Medicine, KMC, Manipal University, Manipal 576104, India

## Abstract

*Introduction*. Recent evidence indicates that clinical examination, for determination of fetal head position, is subjective and inaccurate. Present study was aimed to compare transabdominal ultrasound for fetal head position with vaginal examination during first stage of labor. *Material and Methods*. This prospective study was performed at a tertiary center during a two-year period. Before or after clinically indicated vaginal examinations, transverse suprapubic transabdominal real-time ultrasound fetal head position assessment was done. Frequencies of various ultrasound depicted fetal head positions were compared with position determined at vaginal examination. *Results*. In only 31.5% of patients, fetal head position determinations by vaginal examinations were consistent with those obtained by ultrasound. Cohen's Kappa test of concordance indicated a poor concordance of 0.15. Accuracy of vaginal examination increased to 66% when fetal head position at vaginal examination was recorded correct if reported within +45° of the ultrasound assessment. Rate of agreement between the two assessment methods for consultants versus residents was 36% and 26%, respectively (*P* = 0.17). *Conclusion*. We found that vaginal examination was associated with a high error rate in fetal head position determination. Data supports the idea that intrapartum transabdominal ultrasound enhances correct determination of fetal head position during first stage of labor.

## 1. Introduction

Digital vaginal examination has been the accepted standard to monitor labor. However, recently evidence is indicating that clinical examination (abdominal and vaginal) for the determination of fetal head position is subjective and inaccurate [1]. Furthermore, repeated vaginal examinations can be uncomfortable and associated with risk of introducing infections. Ultrasonography is noninvasive and has been found to be more accurate for assessing position of the fetal head, during labor [2, 3]. Recent studies by Sherer et al. [4], Chou et al. [5], Dupuis et al. [6], and Zahalka et al. [7] have shown that ultrasound scanning is a quick and efficient way of increasing the accuracy of the assessment of fetal head position during the second stage of labor. We would also like to highlight that ultrasound determination of fetal head may allow safe instrumental delivery if required in second stage.

In the present study we aimed to compare ultrasound assessment of fetal head position with vaginal examination during first stage of labor in our setup.

## 2. Material and Methods

This prospective study was carried out in the labor ward at a tertiary care center during a two-year period. The study protocol was approved by institutional ethics committee. All enrolled women provided written informed consent for participation. A total of 165 women with normal singleton, cephalic-presenting fetuses between 35 and 41 weeks' period of gestation with cervical dilatation ≥3 cm were recruited for the study.

The gestational age was determined on the basis of the last menstrual period and a reliable menstrual history, and/or an ultrasound examination before 16 weeks of gestation. Both intact and ruptured membranes were included in this study. Women with multiple pregnancy and women attempting vaginal birth after cesarean delivery were excluded from the study.

Clinically indicated vaginal examinations were performed by attending resident doctor or consultant randomly. The classic method of palpation of the sagittal suture and fontanelles and their location in relation to the maternal pelvis was used to determine fetal head position. Head position was classified as occiput anterior (OA), occiput posterior (OP), left or right occiput transverse (LOT or ROT), left or right occiput anterior (LOA or ROA), or left or right occiput posterior (LOP or ROP).

**Figure 1:**
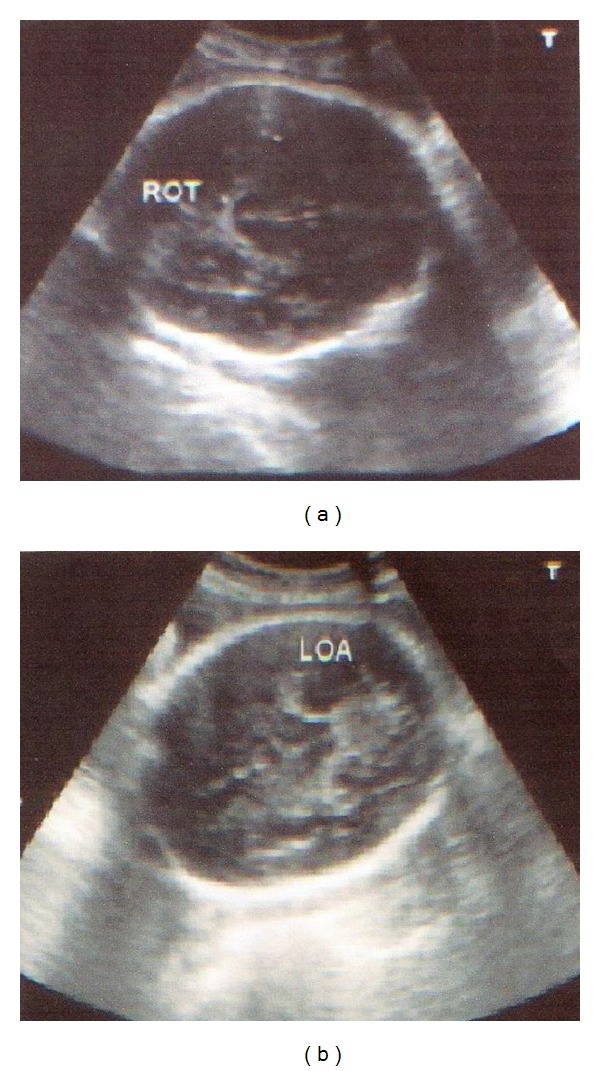
Transverse suprapubic transabdominal real-time ultrasound fetal head position assessment: (a) right occipitotransverse and (b) left occipitoanterior position.

Before or after this vaginal examination, transverse suprapubic transabdominal real-time ultrasound fetal head position assessment was performed by a senior consultant, utilizing an ultrasound unit (Toshiba). During this procedure the ultrasound transducer was first placed transversely in the suprapubic region of the maternal abdomen. Next the fetal spine was demonstrated in its sagittal plane and traced from the fetal thorax to the occiput. Ultrasound depiction of fetal head position was performed utilizing midline intracranial structures (cavum septum pellucidum, falxcerebri, thalami, and cerebellar hemispheres) and anterior and posterior cranial structures (orbit, nasal bridge, and cervical spine). Fetal head position was classified to one of the above-mentioned eight positions (similar to vaginal examination) (Figures 1(a) and 1(b)). The landmarks depicting fetal position were the fetal orbits for occipitoposterior position, the midline cerebral echo for occipitotransverse position, and cerebellum or occiput for occipitoanterior position. While doing an ultrasound assessment, the examiner was blinded off the vaginal examination findings and vice versa.

Frequencies of the various ultrasound depicted fetal head positions were noted. These ultrasound depicted positions were compared with position determined at vaginal examination. With progressive descent of fetal head, internal rotation occurs. Keeping this in mind further analysis of data was done. In this analysis, vaginal fetal head position determination occurring within +45° arc of the respective ultrasound assessment was designated as correct.

Statistical analysis: the degree of agreement between the two examination methods was analyzed by using Cohen's Kappa test. Kappa value < 0.2 was considered to have poor concordance.

## 3. Results

**Figure 2:**
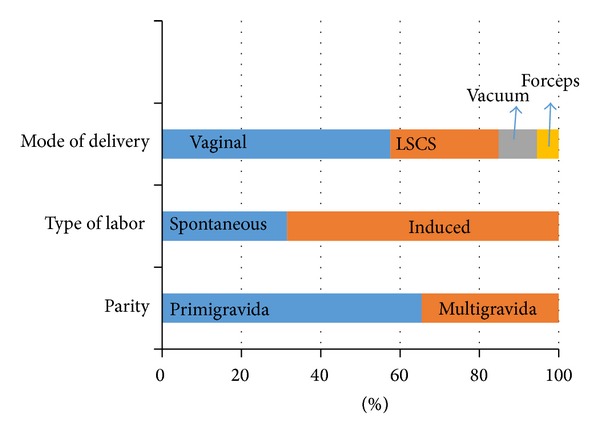
Parity, labor, and delivery details of the population studied.

A total of 165 women were found to be eligible to participate in the study. Demographic characteristics of the population studied are depicted in Table 1 and Figure 2. Mean maternal age was 27.1 + 3.38 years with a mean gestational age of 38.7 + 1.1 weeks. Around two-thirds (65.5%) of these women were primigravida and 34.5% were multigravida. Among these, 31.5% had spontaneous onset of labor while 68.5% were induced for obstetric indications.

Vaginal examinations were carried out by consultants in 47% of women (*n* = 79) or by junior residents with two years (*n* = 31) and senior residents with 3 years of experience in obstetrics and gynecology (*n* = 55). As mentioned earlier before or after this vaginal examination, transverse suprapubic transabdominal real-time ultrasound fetal head position assessment was performed by a consultant. All ultrasound assessments were successful and yielded interpretable fetal head position determinations. Frequencies of the various ultrasound depicted fetal head positions compared with vaginal examinations are presented in Table 2. In only 31.5% of patients (*n* = 52), fetal head position determinations by vaginal examinations were consistent with those obtained by transabdominal ultrasound. Cohen's Kappa test of concordance indicated a poor concordance of 0.15. Vaginal examinations differed by 180° in comparison with the respective ultrasound examinations in 7.8% (*n* = 13) of cases.

With progressing descent of the fetal head, internal rotation occurs and the fetal head which was in LOT may become either in LOA or DOA during vaginal examination. Vaginal fetal head position determinations occurring within +45° arc of the ultrasound assessments of LOT were designated as correct. Twenty-seven patients were in LOA by vaginal examination. This increased the accuracy of vaginal examination in LOT position to 77% (*n* = 47). Cohen's Kappa test of concordance in this comparison modality was 0.59 (moderate concordance). Fetal head which was in ROT as the labor progresses may become either in ROA or DOA during vaginal examination. Vaginal fetal head position determinations occurring within +45° arc of the ultrasound assessments of ROT were designated as correct. Fourteen patients were in ROA by vaginal examination. This increased the accuracy of vaginal examination to 63% (*n* = 24). Cohen's Kappa test of concordance in this comparison modality was 0.53.

Comparison of vaginal examination findings performed by consultants and residents with ultrasound position is shown Tables 3, 4 and 5. Rates of agreement between the two methods for consultants versus residents were not significantly different (*P* = 0.17).

## 4. Discussion 

Accurate intrapartum determination of fetal head position is considered important for the management of both normal and abnormal labor. Few studies have reported on the use of ultrasound scanning for the evaluation of fetal occiput position in first stage of labor. Studies [1–3] found an error rate of 50% to 76% with vaginal examination when ultrasound examination findings were used as the gold standard. Our results indicate that vaginal examination was inaccurate in defining the fetal position during first stage of labor in up to 69% of cases. When vaginal examination findings within +45° of the ultrasound assessment were assigned as correct, the rate of error decreased to 34%, which is still a considerably high error rate. In 7.8% (*n* = 13) of cases vaginal examinations differed by 180° from the sonographically depicted fetal head position. This demonstrates the examiners correct identification of the sagittal suture, yet incorrect designation of the anterior and posterior fontanels. The reason for this error may be failure to examine the patient abdominally before doing a vaginal examination. If the fetal back is correctly identified during abdominal examination and correlated with vaginal examination, the accuracy of vaginal examination can be improved.

Results related to the experience of the obstetrician in determining the position of the fetal head are conflicting. We did not see a significant difference between consultant's and resident doctor's ability to determine position by vaginal examination, whereas Zahalka et al. [7] found that, with the ±45° analysis modality, vaginal examinations performed by consultants were significantly more consistent with ultrasound than were vaginal examinations performed by senior residents (58% versus 33%, resp., *P* = 0.02).

In our cohort, 60% of the fetal heads were in occiput transverse location. Of note, occipitoposterior position was observed in 12% of women in first stage of labor. This was in accordance with other studies [8]. We believe that persistent occiput posterior forms a special risk group for operative delivery. Therefore, early diagnosis could help obstetricians to provide woman with additional information about the need for operative delivery. Specifically accurate assessment of fetal head position is the most important determinant of successful and safe instrumental delivery. Studies performed by Akmal et al. [9] and Wong et al. [10] were able to demonstrate that ultrasound scanning of the fetal head position should be performed routinely before instrumental delivery. In conclusion, we found that vaginal examination was associated with an overall high rate of error in fetal head position determination. These data support the idea that intrapartum application of transabdominal ultrasound significantly enhances correct determination of fetal head position during first stage of labor.

## Figures and Tables

**Table 1:** Demographic characteristics of study population.

Characteristics	Mean ± SD	Range
Maternal age (years)	27.18 ± 3.82	18–38
Height (cm)	155.88 ± 5.31	143–169
Weight (kgs)	61.20 ± 9.75	40.50–97.22
BMI (kg/m^2^)	25.21 ± 3.81	18.49–38.94
Gestational age (weeks)	38.72 ± 1.14	36–41
Birth weight (g)	2967.30 ± 370.1	1880–4160

**Table 2:** Comparison of the fetal head position by ultrasound and vaginal examination.

Fetal head position	Actual number diagnosed by ultrasound *N* = 165 (%)	Agreement in vaginal examination *N* (%)	Other findings in vaginal examination (%)
LOT	61 (37.0)	20 (32.8)	LOA: 27 (44.3), DOA: 02 (2.2), ROA: 05 (8.2), ROT: 05 (8.2), LOP: 02 (3.3)
ROT	38 (23)	10 (26.3)	ROA: 14 (36.8), LOA: 07 (18.4), LOT: 03 (7.9), ROP: 03 (7.9), DOA: 01 (2.6)
LOA	26 (15.8)	12 (46.2)	LOT: 08 (30.8), ROT: 03 (11.5), ROA: 02 (7.7), ROP: 01 (3.8)
ROA	18 (10.9)	03 (16.7)	ROT: 09 (50.0), LOA: 05 (27.8), LOT: 01 (5.6)
LOP	11 (06.7)	01 (09.1)	LOT: 05 (45.5), ROA: 03 (27.3), LOA: 01 (9.1), ROT: 01 (9.1)
ROP	10 (06.1)	10 (06.1)	LOA: 02 (20.0), ROT: 01 (10.0), ROA: 01 (10.0)
DOA	01 (00.6)	00 (00.0)	LOA: 01 (100)

Total	165 (100)	52 (31.51)	

**Table 3:** Comparison of the fetal head position by ultrasound and vaginal examination done by consultant (*N* = 79).

Fetal head position	Actual number diagnosed by ultrasound *N* = 79 (%)	Agreement in vaginal examination *N* (%)	Other findings in vaginal examination (%)
LOT	21 (26.6)	08 (38.1)	LOA: 07 (33.3), ROA: 03 (14.3), LOP: 02 (9.5), ROT: 01 (4.8)
ROT	24 (30.4)	02 (18.2)	ROA: 07 (29.2), LOA: 04 (16.7), LOT: 01 (4.2), ROP: 03 (12.5), DOA: 01 (4.2)
LOA	05 (06.3)	01 (20.0)	LOT: 05 (35.7), ROT: 1 (7.1), ROA: 1 (7.1), ROP: 1 (7.1)
ROA	11 (13.9)	02 (18.2)	ROT: 07 (63.6), LOA: 02 (18.2)
LOP	05 (06.3)	01 (20.0)	LOT: 02 (40), ROA: 02 (40)
ROP	04 (5.1)	04 (100)	—

Total	79 (100)	29 (36.7 )	

**Table 4:** Comparison of the fetal head position by ultrasound and vaginal examination done by senior residents.

Fetal head position	Actual number diagnosed by ultrasound *N* = 55 (%)	Agreement in vaginal examination *N* (%)	Other findings in vaginal examination (%)
LOT	28 (50.9)	09 (32.1)	LOA: 14 (50.0), DOA: 01 (3.6), ROA: 01 (3.6), ROT: 03 (10.7)
ROT	09 (16.4)	01 (11.1)	ROA: 05 (55.6), LOT: 01 (11.1), LOA: 02 (22.2)
LOA	04 (07.3)	02 (50.0)	LOT: 01 (25.0), ROA: 01 (25.00)
ROA	05 (9.1)	00 (00.0)	ROT: 02 (40.0), LOA: 02 (40.0), LOT: 01 (20.0)
LOP	05 (9.1)	00 (00.0)	LOT: 02 (40.0), ROA: 01 (20.0), ROT: 01 (20.0), LOA: 01 (20)
ROP	03 (5.5)	01 (33.3)	LOA: 01 (33.3), ROA: 01 (33.3)
DOA	01 (1.8)	00 (00.0)	LOA: 01 (100)

Total	55 (100%)	13 (23.63)	

**Table 5:** Comparison of the fetal head position by ultrasound and vaginal examination done by junior residents.

Fetal head position	Actual number diagnosed by ultrasound *N* = 31 (%)	Agreement in vaginal examination *N* (%)	Other findings in vaginal examination (%)
LOT	12 (38.7)	03 (25.0)	LOA: 06 (50.0), DOA: 01 (8.3), ROT: 01 (8.3), ROA: 01 (8.3)
ROT	05 (16.1)	01 (20.0)	ROA: 02 (40.0), LOA: 01 (20.0), LOT: 01 (20.0)
LOA	08 (25.8)	04 (50.0)	LOT: 02 (25.0), ROT: 02 (25.0)
ROA	02 (6.5)	01 (50.0)	LOA: 01 (50.0)
LOP	01 (3.2)	00 (00.0)	LOT: 01 (100)
ROP	03 (9.7)	01 (33.3)	LOA: 01 (33.3), ROT: 01 (33.3)

Total	31 (100)	10 (32.25)	
